# A Systematic Review of Acute Thoracic Aortic Dissections in Africa—The Need for a Registry

**DOI:** 10.1055/s-0042-1757797

**Published:** 2022-12-20

**Authors:** Anthony Yip, Elena Libhaber, Penelope Nam, Robert Kleinloog, Lorenzo Rampini, Catherine Hosking

**Affiliations:** 1 Department of Cardiology, Life Fourways Hospital, Johannesburg, South Africa; 2 School of Health Sciences, University of Witwatersrand, Johannesburg, South Africa; 3 Department of Cardiothoracic Surgery, Ethekweni Heart Centre and Busamed Gateway Private Hospital, Durban, South Africa; 4 Department of Cardiothoracic Surgery, Olivedale Clinic, Johannesburg, South Africa; 5 Department of Anesthesiology, Morningside Medi-Clinic, Johannesburg, South Africa

**Keywords:** thoracic aortic dissections, Type Stanford A dissection, Type Stanford B dissection, Africa

## Abstract

In this systematic review, the available literature on the presentation and management of acute thoracic aortic dissections in Africa is examined. Though Africa has 17% of the world population, it accounts for approximately 1% of the available literature with much of our understanding coming from registries arising from the developed world, such as the International Registry of Acute Aortic Dissection. The literature from the African continent consists mainly of case reports, small case series, and few original studies. Case reports make an important contribution to our understanding of uncommon conditions but can skew our understanding of aortic dissections in this region by describing unusual presentations and management. In this review, we describe the available studies retrieved from large medical databases (Medline and Health Management Information Consortium) and motivate the need for national registries to provide a more accurate appreciation of the scope of the problem on this continent.

## Introduction

Acute thoracic aortic dissections are relatively rare with an estimated incidence of 2.53 per 100,000 but with high morbidity and mortality. 1 Presentations can vary from chest, back, or abdominal pain; syncope; organ malperfusion syndromes; to cardiac tamponade and death. 2 Aortic dissections have a long history with the first fully described case being the sudden death of King George II of Great Britain in 1760. Meaningful treatment for this condition was only developed in the 1950s and 1960s with the pioneering work of the famed cardiothoracic surgeons, DeBakey et al. 3 4

Our understanding of aortic dissections has grown over the past two to three decades: Clinicians appreciate the importance of early surgery with ascending aortic dissections and the role of endovascular stenting in descending aortic dissections (where medical management was previously the standard of care). In addition, we have a better grasp of the role of inherited connective tissue disorders including Marfan's, Ehler's–Danlos, and the Loeys–Dietz syndromes and the genetics involved in nonsyndromic, familial aortic dissections. 5 6

The knowledge gained is helping physicians prevent dissections in at-risk populations. Imaging modalities and guidelines on measurements are improving and becoming more standardized. 7 From the emerging genetic data, dedicated aortic centers are refining surgical guidelines to prevent dissections in patients with high-risk genes. 5 8 Recommended aortic diameters at which prophylactic surgery is offered are tailored to one's genetic profile. 8 Even the use of aortic diameter alone as a measure of risk is being challenged by examining the role of aortic wall thickness. 9

Many of our insights on aortic dissections, which have improved our understanding and management of this disease, have come from large multinational registries such as the International Registry of Aortic Dissection (IRAD) which is now over 20 years old. 10 It is a large registry including over 7,300 patients from around 51 centers from 12 countries from North America, Europe, Asia, and Australasia (iradonline.org). Similarly, the Nordic Consortium for acute Type A aortic dissections includes eight centers from the Nordic countries of Denmark, Finland, Iceland, and Sweden. 11 Despite the value of these registries, it is noticeable that they do not include any centers from the African continent. This gap in our understanding of how aortic dissections present and are managed in Africa needs to be addressed.

## Objectives

The purpose of this systematic review was to facilitate our understanding of how acute thoracic aortic dissections present and are managed in South Africa and the rest of Africa.

We aimed to address the following questions on the presentation of this condition:

- What are the most common symptoms and clinical signs on presentation?
- What are the underlying etiologies described—are they different from what is described in the international literature?
- Do patients present later?
- How have patients been managed—is there a greater use of medical therapy versus surgery because of resource limitations?

We audited the available literature on the management of aortic dissections in Africa to determine whether it addressed the following issues:

- Surgery versus medical management. 12
- Open surgery versus endovascular stenting. 13 14
- β blockers versus other antihypertensive agents. 15
- The use of angiotensin receptor blockers versus other antihypertensives (there has been speculation that the angiotensin II receptor blockers losartan reduces the expression of transforming growth factor β which has been implicated in the growth of aneurysms). 16

## Materials and Methods

A systematic review was initiated in October 2020. The intention was to evaluate available clinical studies and perform a meta-analysis, if appropriate, on the management of acute thoracic aortic dissections in Africa.

The review was conducted following the principles outlined in the 2009 Preferred Reporting Items for Systematic Reviews and Meta-Analyses guidelines 17 by two independent doctors with clinical insight into the conditions. The established medical databases, PubMed/Medline and Health Management Information Consortium, were used to do a comprehensive literature search on October 31, 2020. Details of the protocol for this systematic review were registered on the International Prospective Register of Systematic Reviews (PROSPERO) and can be accessed at: *www.crd.york.ac.uk/PROSPERO/display_record.asp?ID=CRD42021268459* .

**Fig. 1:**
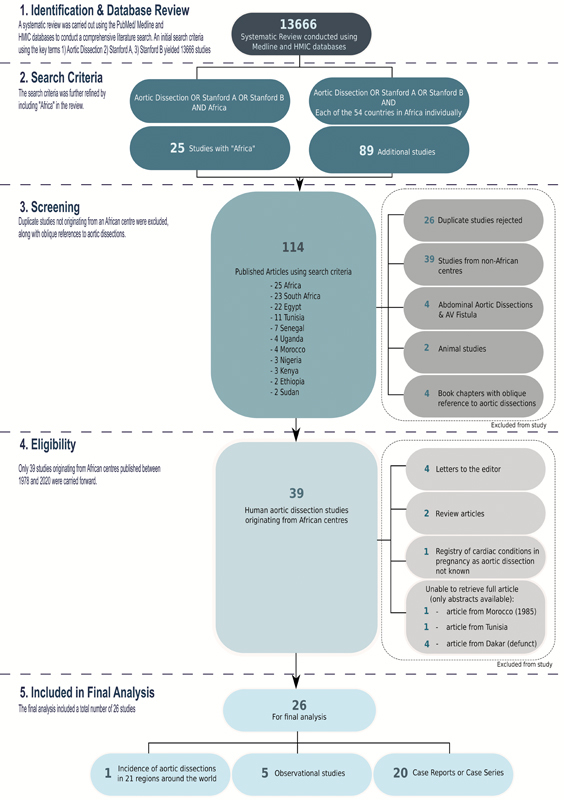
Flow chart depicting process of aortic dissection systematic review within Africa.

Studies used for the systematic review included only published studies in peer-reviewed journals such as clinical trials, previous systematic reviews, and case reports. Foreign language articles were included. A flow chart depicting the systematic review process is illustrated in Fig. 1 .

An initial search of the two databases used the key terms mentioned below:

- Aortic dissection.
- Stanford A dissection.
- Stanford B dissection.

This search yielded 13,666 studies in total.

Thereafter, the same key terms were used as follows:

- Aortic dissection.
- Stanford A dissection.
- Stanford B aortic dissection.
- Africa.

Twenty-five studies were yielded in combining the databases.

The search was then repeated using the following terms:

- Aortic dissection.
- Stanford A dissection.
- Stanford B dissection.
- The name of each of the 54 separate countries in Africa as listed by the United Nations (UN.org).

An additional 89 studies were yielded in this third step.

Using this strategy, 114 published articles were yielded ( Fig. 1 ). Duplicate articles, studies that did not originate from an African center, studies of abdominal dissections, animal studies, and book chapters (with oblique references to aortic dissections) were excluded. Only 39 studies originating from African centers, published between 1978 and 2020, were, thus, obtained ( Fig. 1 ).

From these 39 studies, we further excluded: four letters to the editor, two article reviews (one was a review on the current knowledge and management of aortic dissections not specific to Africa, and a review of chest pain syndromes with an indirect reference to aortic dissections), and a registry of cardiac disease in pregnancy (as the underlying cause of the three patients with aortopathies in African countries was not known). A further six were excluded as only abstracts were available (four were from the now defunct *Dakar Medical Journal* which stopped publishing in 2008, one was from *Tunisia Medicale* with articles from their archives before 2004 unavailable, and one from the *Moroccan Medical Journal* from 1985 was also unavailable).

Of the remaining 26 studies, carried through to the final analysis:

- There were five observational studies: three interventional studies (one of surgery in Stanford A patients, 18 one of endovascular stenting in Stanford B patients, 19 and one of cardiac bypass surgery in human immunodeficiency virus [HIV]-positive patients 20 ) and two postmortem studies looking at causes of sudden cardiac death in a general population and among athletes. 21 22
- We included one study which arose from our search of articles from “Africa” which was written by an author from Vanderbilt University in the United States. 23 It was a retrospective record review which looked at the incidence of aortic dissections and aortopathies in 21 regions around the world based on ICD 10 diagnostic codes in records provided. The study included regions in Africa and was the only available article in the literature that provided a global perspective of the incidence of aortic dissection.
- There were 20 case reports or case series. 24 25 26 27 28 29 30 31 32 33 34 35 36 37 38 39 40 41 42 43

**Fig. 2:**
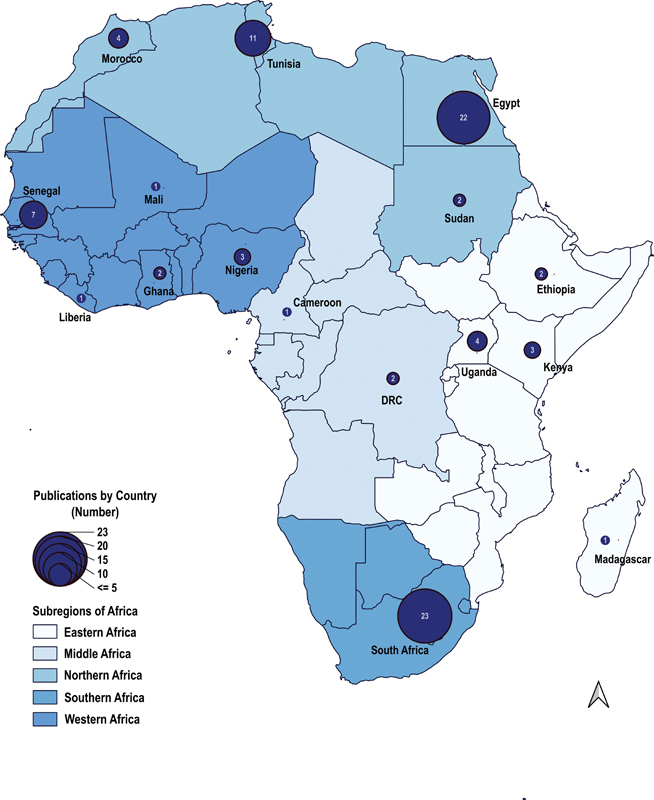
Aortic dissection publications within Africa.

Available demographic and clinical data were collected and captured on an Excel Spreadsheet (see Fig. 2 ).

A summary of the five observational studies was tabulated ( Table 1 ) according to The Strengthening of Observational Studies in Epidemiology (STROBE) recommendations 44 in optimizing the reporting for these types of studies. Similarly, the CARE (Case Report) guidelines 45 for reporting were used as a template in evaluating and tabulating the case reports we included.

**Table 1:** Demographics, ethnicity, etiology and presenting symptoms of patients with acute aortic dissection within Africa

Patient characteristics	Total articles a (number)	Stanford A (number)	Stanford B (number)	Conditions mimicking dissection (number)
	**25**	**17**	**6**	**2**
Age (average in years)	47.2	41.2	52.3	47.5
Male	17	10	6	1
*Ethnicity* :				
Black	5	4	1	–
Not stated	17	12	4	1
Other	3	1	1	1
*Etiology* :				
Hypertension	13	8	5	–
Atherosclerosis	0	–	–	–
Marfan's	1	1	–	–
Takayasu	1	–	1	–
Other	2	–	–	2
*Presentation* :				
Chest pain	18	12	4	2
Abdominal pain	2	1	1	–
Heart failure and hemopericardium	2	2	–	–
Paraplegia	2	1	1	–
Unknown	2	2	–	–

a Excluding the one global incidence report of aortic disease by Sampson et al. 23

Our initial objective was to conduct a meta-analysis of outcomes in both Stanford A and Stanford B dissections using either surgical or medical management, and open surgery or endovascular stenting if the studies had complete and reliable data. Meta-analysis comparing medication regimens used was not possible as data were inhomogeneous. The observational studies of intervention included for analysis were also inhomogeneous and not suitable for meta-analysis. No randomized control trials were found in our search.

## Results

### Distribution and Types of Studies

Of the 26 studies from African medical centers which were included for analysis, 19 were in English, and seven were in French. Most studies, 20, were case reports, 5 were observational studies (2 of the 5 observational studies were postmortem), and one was a global review which included regions in Africa.

### Epidemiology

In these studies, clinical information was often incomplete. The background clinical characteristics were extracted, when available, from the 20 case reports and the five observational studies ( Tables 1 , 2 , 3 ). As studies were not uniform, each characteristic in the Tables is represented according to the number of articles that featured it ( Table 1 ).

**Table 2:** Five observational studies

	Study/country/language/publication date	Study design	Objectives	Patient characteristics	Management	Outcome	Key points	Sample size
*Stanford A* :								
1	von Oppell et al 18 /South Africa/English/2002	Observation, retrospective, case-control, record review of 41 consecutive patients with ascending aortic dissection who had surgery	Compare outcome of patients who had dissection repaired with gelatin-resorcin-formaldehyde -glutaraldehyde (GRF) vs. those without	Mean age 61.3 yeas with male preponderance in GRF group	19 acute dissections: aortic valve resuspension and GRF glue; 22 dissections (9 acute and 13 chronic): aortic valve resuspension without GRF glue	24.4% operative mortality, 52.1% 5-year survival in GRF group vs. 55.6% 5-year survival non-GRF group. Higher complications in GRF group	The use of GRF glue for aortic valve resuspension and valve repair was associated with poorer outcomes	41
2	Blyth et al 20 /South Africa//English/2006	Observational, retrospective, cross-sectional, record review of 49 HIV patients going for cardiac surgery	To determine whether the coexistence of HIV in an era before antiretroviral therapy was associated with poorer surgical outcomes	17 male, 32 female, age 17–67 years with average of 33 years	Only one patient with aortic dissection included in this study	Patient with aortic dissection did not have a CD4 recorded, survived to discharge but was lost to follow-up	Surgical outcomes were similar to uninfected in early HIV. Cardiopulmonary bypass did not accelerate progression of disease	49
3 a	Allouche et al 21 /Tunisia/French/2013	Observational, retrospective, cross-sectional, record review of 32 postmortem cases of sudden death in athletes	To determine causes of sudden death in athletes	84% male with average age of 33.16 years	Unknown as postmortem study	One patient identified with aortic dissection (Marfan's syndrome)	Aortic dissection as a cause of sudden death in athletes	32
4 a	Mesrati et al 22 /Tunisia/French/2017	Observational, retrospective, cross-sectional, record review of 361 postmortem cases of sudden death	To determine causes of sudden death in a single region	Incidence of sudden cardiac death 9 per 100,000, male predominance, mean age 55.75 years	Unknown as poststudy	1.3% of patients identified with aortic dissection	Aortic dissection as a cause of sudden cardiac death in a population	361
*Stanford B* :								
5	Soliman et al 19 /Egypt/English/2018	Observational, prospective, cohort study following up 30 patients with uncomplicated Stanford B dissections or descending aneurysms who had endovascular stenting	To determine mortality or complications associated with EVAR	24 men and 6 women with mean age 59 years ± 8. 24 patients with dissection and 6 aneurysms, 90% hypertension, 83.3% smokers, 60% dyslipidemia, 16.7% diabetic, and 26.7% CAD	Of 24 dissections 17 were stented alone, 7 had hybrid procedure	10% mortality (two patients with endoleak) complications occurred in subacute and chronic cases	Early stenting of Stanford B is a feasible technique with better outcome then later stenting	30

Abbreviations: CAD, coronary artery disease; EVAR, endovascular aneurysm repair; HIV, human immunodeficiency virus.

a No clinical data available.

**Table 3:** Case reports

Study/country/language/publication date	Patient characteristics					Medication, surgery, or stent	Outcome	Key points	Missing data
	Age (y)	Gender	Ethnicity ^a^	Clinical presentation	Etiology				
*Stanford A* :									
von Oppell et al 24 /South Africa/English/1995	60 and 57	60-y female and 57-y unknown	N/S	Chest pain (both patients). Fistula from aorta to right atrium in one patient.	Hypertension in both	Patients had aortic grafts with the use of gelatin–resorcin–formaldehyde–glutaraldehyde (GRF) glue to repair dissection layers	Both survived to discharge but required permanent pacemakers	GRF devitalizes tissue with conduction disturbances possible	Gender of 52-y-old not known, ethnicity, medication given
Abdou et al 25 /Egypt/English/2011	58	Male	N/S	Chest pain with anterior myocardial infarction. Diagnosed on echocardiography	Atherosclerotic	Coronary stent to right coronary ostium.	Survived to discharge	Iatrogenic ascending aortic dissection can occur with coronary interventions	Ethnicity, center of procedure
Mesrati et al 26 /Tunisia/English/2019	45	Female	N/S	Retrosternal chest pain radiating to the epigastrium	Unknown	Treated as an ulcer with proton-pump inhibitors	Died after discharge home. Diagnosed on autopsy.	Thoracic aortic dissections extending to abdominal aorta can mimic peptic ulcer disease	Ethnicity
Mleyhi et al 27 /Tunisia/English/2018	61	Male	N/S	Abdominal pain. H/D compromised. Stanford A with abdominal aneurysm—aortocaval fistula on CT	Hypertension	Endovascular stent planned	Rapid deterioration. Died before surgery	Image case. Aortic dissection occurring after previous aortic valve replacement	Medication, ethnicity
Abderrahim et al 28 /Tunisia/English/2020	15	Female	N/S	Painful left mass from a carotid paraganglioma. Stanford A	Hypertension	Had no surgery	Died at home. Hemopericardium	Carotid paraganglioma as a rare cause for an aortic dissection from a catecholamine surge	Ethnicity
Abdelmoula et al 29 /Tunisia/English/2018	16	Female	N/S	Chest pain	Turner's syndrome with bicuspid valve, Coarctation Hypertension	Surgical repair at 5 y of age after acute Stanford A dissection	Survived dissection and was 16 y at time of genetic studies	Turner's syndrome as a cause for dissection. Left sided outflow tract anomalies may be linked to X-chromosome	Medication, ethnicity
Hdiji et al 30 /Tunisia/English/2016	70	Male	Arab	Painless paraplegia with urinary retention	None previously known	No surgery. Diagnosis made on autopsy	Rapid deterioration with cardiogenic shock; death from hemopericardium	Acute painless paraplegia as a rare presentation for Stanford A dissection	No antemortem cardiac imaging reported
Sarr et al 31 /Senegal/English/2017	24*	Two male who had aortic dissections	Black	Two family members with aortic dissection; Chest pain. Diagnosis on CT scan	Marfan's syndrome	One patient had Bentall procedure, second patient did not have surgery for lack of funds. All treated with β blocker	Patient with surgery recovered well. Unoperated patient alive after 1 y.	Marfan's syndrome with one family showed a high incidence of aortopathy	–
Moh et al 32 /Ivory Coast/French/2018	35	Male	Black	Chest pain, cough, fever, and dyspnea with diagnosis on transesophageal echo	HIV and tuberculosis	Medically treated with antiretroviral and antituberculosis treatment	Alive 8 y after dissection despite no surgery	HIV and possibly a reconstitution inflammatory syndrome after instituting ARV as a cause for aortopathy	–
Masinarivo et al 33 /Madagascar/French/2017	52	Female	N/S	Chest pain and dyspnea. Diagnosis on CT scan. ECG-RBBB with S1, Q3, T3 suggesting pulmonary embolus	Hypertension, smoker and obese	Medically treated as surgery facilities unavailable	Survived to discharge	Aortic dissection mimicking clinical and ECG pattern of pulmonary embolus	Image case. Medication, ethnicity
Benbouchta et al 34 /Morocco/English/2020	62	Male	N/S	Chest pain, shortness of breath and diaphoresis. ECG showed ST segment in inferior leads. Diagnosis on echo	Hypertension	Bentall procedure with valve sparing and interposition graft. Antihypertensives given	Survived to discharge	Aortic dissection with right coronary involvement may present as an inferior STEMI	Medication, ethnicity
Swift et al 35 /Liberia/English/2019	Middle aged	Male	Black	Chest pain, palpitations, and diaphoresis. Diagnosis on echocardiography.	Unknown	Surgical repair after transfer to Ghana	Survived to discharge	First aortic dissection successfully operated from Liberia. Resource poor country, hence, surgery in Ghana	Medication, exact age not given
Bouramoue et al 36 /DRC/French/2001	40 a	Three males; three females	Black	Chest pain	Hypertension	Two patients operated on, but four treated medically	Two operated patients survived. The remaining four died	High association with hypertension and query the importance of Takayasu's as a cause in the region.	–
Stanford B									
Azzabi et al 37 /Tunisia/French/2009	75	Male	N/S	Heart failure, pleural effusion, heart block and DIC. Chronic Stanford B dissection with calcified dissection flap on CT scan	Hypertension	Medically treated no surgery as patient refused treatment against medical advice	Died at home 4 d after leaving hospital	Disseminated intravascular coagulation as an unusual presentation for chronic dissection	Ethnicity
Siddo et al 38 /Senegal/French/2014	67	Male	N/S	Chest pain with a hemothorax. Stanford B dissection diagnosed on CT scan	Hypertension	Medically treated with β blocker, atenolol, and analgesia	Died 48 h after diagnosis	Hemothorax as a presentation of a Stanford B dissection	Ethnicity
Zaghdoudi et al 39 /Uganda/French/2014	67	Male	Maghrebin tribe	Abdominal pain with Stanford B diagnosed on CT scan	Takayasu's	Medically treated with β blocker and steroids for Takayasu's arteritis	Survived to discharge	Takayasu's arteritis as a cause for a Stanford B dissection	–
Ogun et al 40 /Nigeria/English/2004	46	Male	N/S	Parasternal chest pain to back, sudden onset paraparesis. Diagnosis made clinically. No imaging	Hypertension	Medically treated with β blocker, propranolol, calcium channel blocker, amlodipine, pentazocine and prednisolone. No surgery or stenting available	Died 8 d after admission	Anterior spinal syndrome as a presentation of Stanford B dissection	Method used to confirm diagnosis not clear as imaging not performed
Waweru et al 41 /Kenya/Language/English/2016	57	Male	Black	Progressive chest pain. Diagnosis on CT. Developed abdominal pain post stenting	Hypertension	Endovascular stent inserted	Ongoing abdominal pain despite stenting. After steroids, inflammation, settled—discharged	Postimplantation syndrome from local inflammation because of endovascular stent implantation	–
*Intramural hematoma mimic Stanford B* :									
Elsamman et al 42 /Egypt/English/2019	30	Male	N/S	Chest pain and dysphagia. CT chest- para-aortic hematoma compressing esophagus mimic Stanford B dissection	Behcet's disease	Endovascular stent inserted in descending aorta	Symptoms relieved and patient survived to discharge	Behcet's causing an intra-aortic hematoma with a Stanford B-like appearance on CT and dysphagia aortica	Medication, ethnicity
*Leiomyosarcoma descending aorta mimic Stanford B* :									
Golli et al 43 /Tunisia/English/2000	65	Female	Caucasian	Chest pain. CT chest showed thickening of aorta with 7-cm periaortic mass—mimic Stanford B	Leiomyosarcoma of the aorta	Surgical resection and graft with diagnosis confirmed after surgery and histology	Died 3 mo after surgery	Leiomyosarcoma of the aorta mimicking Stanford B dissection	–

Abbreviations: ARV, antiretrovirals; CT, computed tomography; DIC, disseminated intravascular coagulation; ECG-RBBB; electrocardiogram—right bundle branch block; HIV, human immunodeficiency virus; N/S, not stated; STEMI, ST-elevation myocardial infarction.

a Average age.

Trends in Africa were in keeping with our current understanding of aortic disease from established registries such as IRAD, with a male preponderance, an association with hypertension, a higher proportion of Stanford A dissections, and chest pain being the most common initial presentation for dissection ( Table 1 ).

The average age of Stanford A patients was 41.2 years of Stanford B patients was 52.3 years of age, and the average age of total number of patients with thoracic aortic dissections was 47.5 years of age.

Where data were available, patients were predominantly of the Black race and other ethnicities included patients of Arab descent, especially in studies originating from North Africa.

Most of these numbers were not statistically significant.

The true incidence of aortic diseases and aortic dissection in Africa, and its individual countries, are not known. But, in the study by Sampson et al 23 from Vanderbilt University, data from the Global Burden of disease study were taken to estimate the incidence of aortic aneurysm- and aortic dissection-related mortality in different regions in the world from 1990, at the time of their initial study, until 2010.

They found that the global mortality rate per 100,000 population had increased from 2.49 to 2.78. In developed countries, the relative increase over the 20 years was the smallest. In comparison, the relative increase in developing countries, which included central Sub-Saharan Africa, was among the greatest. This was despite the low incidence of aortic diseases which was the result of a “paucity of surveillance data” from these regions. The authors postulate that this epidemiological transition in aortic disease patterns may signal potential challenges for the poorly equipped health care systems of the developing world.

### Etiology

Hypertension was the most frequently associated underlying cause. Other etiologies reported included connective tissue disorders such as Marfan's syndrome, inflammatory conditions such as Takayasu's arteritis, and the HIV.

### Management

From the available case reports, it was surprising that Stanford A dissections were still managed conservatively without urgent surgical intervention. The reasons for this included lack of availability of surgical facilities or surgeons in the admitting center, lack of availability in the country, or rapid deterioration of the patient before surgery.

As expected, Stanford B patients were managed medically. However, one observational study from Egypt included a study group of 24 Stanford B aortic dissection patients that were managed with endovascular aortic replacement (EVAR) in a clinical trial, in a highly specialized center, with good outcomes.

### Outcomes

In the Stanford A patients, survival was poor without surgery. The case studies that reported survival without surgical intervention were likely reported because of their rarity. Even where surgery was offered, the mortality rate was as high as 24%. 18

No Stanford B patients were offered open surgery, and survival to discharge was 60% for those treated with medication alone. The outcomes of endovascular stenting were shown to be favorable in Stanford B dissections where the patient selection was appropriate. 19

## Discussion

This systematic review on aortic dissections is, to our knowledge, the first of its kind conducted in Africa. It has shown the lack of data on thoracic aortic dissections from this continent. Though we have attempted to be thorough in our review of available studies in Africa, it is likely that a few studies may have been missed. There are other large medical databases such as EMBASE (which was not used because of availability and costs). Nonetheless, we believe this review is a true representation of the lack of studies on this condition originating in Africa: a continent with almost a fifth of the world's population publishing less than 1% of the literature on aortic dissections.

There may be several reasons for this including financial, hospital resources, lack of expertise, and perhaps, a lower incidence of this condition. Regardless of the reasons, we do not think that the available literature accurately reflects the presentation and management of aortic dissections in Africa.

### Publication Bias of Case Reports

The majority of publications were dominated by case reports with few original research articles found from this region. Though randomized controlled trials are the gold standard of clinical studies, it may not be possible to get a sufficient number of subjects over a meaningful period (e.g., 5 years) in relatively rare diseases. Thus, case reports can be invaluable in providing insights into uncommon disorders, at the risk of skewing perceptions due to unusual presentations of this condition.

### Emerging Trends to Be Explored

Despite these limitations, there were interesting trends that emerged in studying the literature. There were some similarities between epidemiology first provided to us by IRAD 20 years ago, and the patterns in our review: a male preponderance, a similar mean age of presentation, an association with hypertension, and a greater number of Stanford A versus Stanford B dissections.

Interesting etiologies that were associated with aortic dissections were the human immunodeficiency virus and rare autoimmune conditions such as Takayasu's arteritis. Are these conditions seen and associated more commonly with dissections on this continent than elsewhere?

There also is a remarkable lack of reporting regarding familial aortic dissections and no identifiable genetic studies in this region. Are familial syndromes rarer in Africa or under-investigated?

### Management of Aortic Dissections in Africa

From available studies, we see that resources appear severely limited in parts of Africa and medical management for Stanford A dissections may be the only option, where the transfer to neighboring countries is required if a patient is to get needed surgery. On the contrary, a well-conducted interventional trial using EVAR in Stanford B patients in Egypt showed that there are centers in continents that are able to offer state-of-the-art treatment to their patients.

However, because of the lack of available original articles and registry data, we may not have an accurate view of how this condition is managed on this continent.

The experience of the authors from their own institutions in South Africa is that the management of Stanford A and Stanford B dissections are more in line with IRAD data using European and U.S.-based guidelines.

## Future Directions and the Need for National Registries

We do not believe that the lack of data in Africa represents a lower incidence of aortic dissections on this continent, but rather an under-reporting of this condition. It is noticeable that IRAD does not include an African center.

There exists a need in Africa to develop a better understanding of how dissections manifest and our management here:

- What are the differences in etiology and management?
- Where are the centers of excellence?
- How can we improve outcomes?

In the study of uncommon conditions such as aortic dissection, the recruitment of patients for a randomized-controlled trial may take a long time and be prohibitively expensive for resource-limited countries. A registry has the potential to be a powerful tool. It allows centers from different countries to pool resources.

In August 2020, a pilot launch of a South African Registry of Acute Aortic Dissections was conducted under the auspices of the South African Cardiothoracic Society. The plan was to create a national registry to better understand and manage this rare but potentially fatal condition. We believe it will provide much needed data and create the environment where centers of excellence may emerge and extend into the rest of Africa.

## Limitations

This systematic review was limited by the limited number of studies with often incomplete clinical data. Statistically meaningful conclusions were often not possible, and no definitive conclusions could be drawn. More high-quality, original studies and registry data showing real-world outcomes in Africa are needed.

## Conclusion

There is a paucity of data on acute thoracic aortic dissections from Africa, with few original studies. Though Africa has 17% of the world's population, it accounts for less than 1% of the published literature on this topic. There are limited high-quality, original articles with a preponderance of case reports.

Though case reports provide useful insights, inherent selection bias may not represent real-world presentation and management on this continent. In a resource-poor region, randomized control trials may be prohibitively expensive and logistically difficult to conduct. National registries may provide the means of pooling useful data across this region for the benefit of patients of this relatively rare but important condition.
